# Insomnia as a mediating therapeutic target for depressive symptoms: A sub‐analysis of participant data from two large randomized controlled trials of a digital sleep intervention

**DOI:** 10.1111/jsr.13140

**Published:** 2020-08-18

**Authors:** Alasdair L. Henry, Christopher B. Miller, Richard Emsley, Bryony Sheaves, Daniel Freeman, Annemarie I. Luik, Donna L. Littlewood, Kate E. A. Saunders, Jennifer C. Kanady, Jenna R. Carl, Michelle L. Davis, Simon D. Kyle, Colin A. Espie

**Affiliations:** ^1^ Big Health Inc. San Francisco California USA; ^2^ Sleep and Circadian Neuroscience Institute Nuffield Department of Clinical Neurosciences University of Oxford Oxford UK; ^3^ Department of Biostatistics and Health Informatics Institute of Psychiatry, Psychology and Neuroscience King's College London London UK; ^4^ Department of Psychiatry University of Oxford Oxford UK; ^5^ Department of Epidemiology Erasmus MC University Medical Centre Rotterdam The Netherlands; ^6^ NIHR Greater Manchester Patient Safety Translational Research Centre University of Manchester Manchester UK

**Keywords:** cognitive behavioural therapy, depression, internet, sleep

## Abstract

Insomnia predicts the onset of depression, commonly co‐presents with depression and often persists following depression remission. However, these conditions can be challenging to treat concurrently using depression‐specific therapies. Cognitive behavioural therapy for insomnia may be an appropriate treatment to improve both insomnia and depressive symptoms. We examined the effects of a fully‐automated digital cognitive behavioural therapy intervention for insomnia (*Sleepio*) on insomnia and depressive symptoms, and the mediating role of sleep improvement on depressive symptoms in participants from two randomized controlled trials of digital cognitive behavioural therapy for insomnia. We also explored potential moderators of intervention effects. All participants met criteria for probable insomnia disorder and had clinically significant depressive symptomatology (PHQ‐9 ≥ 10; *n* = 3,352). Individuals allocated to treatment in both trials were provided access to digital cognitive behavioural therapy. Digital cognitive behavioural therapy significantly improved insomnia (*p* < .001; *g* = 0.76) and depressive symptoms (*p* < .001; *g* = 0.48) at post‐intervention (weeks 8–10), and increased the odds (OR = 2.9; 95% CI = 2.34, 3.65) of clinically significant improvement in depressive symptoms (PHQ‐9 < 10). Improvements in insomnia symptoms at mid‐intervention mediated 87% of the effects on depressive symptoms at post‐intervention. No variables moderated effectiveness outcomes, suggesting generalizability of these findings. Our results suggest that effects of digital cognitive behavioural therapy for insomnia extend to depressive symptoms in those with clinically significant depressive symptomatology. Insomnia may, therefore, be an important therapeutic target to assist management of depressive symptoms.

## 1 INTRODUCTION

Depression is a common disorder that is estimated to affect 9%–20% of adults (Kessler, Petukhova, Sampson, Zaslavsky, & Wittchen, 2012). Despite effective pharmacological and psychological treatments, depression remains the leading cause of disability worldwide (Friedrich, 2017). Insomnia is a promising therapeutic target in depressive disorders because sleep disturbance is a typically co‐morbid characteristic; and because insomnia symptoms often persist with otherwise effective treatment of depression (Nutt, Wilson, & Paterson, 2008). Moreover, meta‐analytic evaluation suggests an aetiological link; individuals with pre‐existing chronic insomnia being twice as likely to develop first episode depression (Baglioni et al., 2011).

Cognitive behavioural therapy (CBT) for insomnia is an effective intervention for insomnia and co‐morbid depressive symptoms (Gebara et al., 2018). Studies of in‐person CBT have shown beneficial effects when delivered standalone or adjunctive to depression treatments (Gebara et al., 2018); however, systemic barriers prevent widespread access to in‐person CBT (Koffel, Bramoweth, & Ulmer, 2018). The evolution of digital CBT formats (web/mobile) address accessibility challenges around managing insomnia (Luik, Kyle, & Espie, 2017). Both guided (Blom et al., 2015; van der Zweerde, Van Straten, Effting, Kyle, & Lancee, 2019) and fully‐automated digital CBT (Christensen et al., 2016; Luik, Bostock, et al., 2017) for insomnia demonstrate similar effects on depressive symptoms as in‐person CBT for insomnia. Nevertheless, the effects of fully‐automated digital CBT on depressive symptoms in participants with clinically significant depression symptomatology have yet to be examined. Although examined in trials of therapist‐delivered CBT (Manber et al., 2016), the mediating role of insomnia on depressive symptoms has also not been investigated using digital CBT. Data from trials of fully‐automated digital CBT provide an opportunity to test this hypothesized relationship between sleep and subsequent depressive symptom improvement in large sample sizes.

In this short report, we examined the effects of digital CBT for insomnia compared with control on insomnia and depressive symptoms, and the mediating role of insomnia using a sub‐sample of participants with insomnia and clinically significant depression symptomatology from two large effectiveness trials. These trials investigated the mediating role of insomnia on daytime functioning (Espie et al., 2019) and mental health following digital CBT (Freeman et al., 2017) in individuals with insomnia. We also explored potential moderators of intervention effects.

## 2 METHODS

In both randomized controlled trials (RCTs), participants scoring < 16 on the Sleep Condition Indicator (SCI‐8), suggestive of probable DSM‐5 insomnia disorder, had been randomized to either *Sleepio*, a homogeneous fully‐automated digital CBT intervention for insomnia, or to a control condition (sleep hygiene education [Espie et al., 2019] or usual care [Freeman et al., 2017]). Both trials used validated measures for insomnia (SCI‐8) and depression (Patient Health Questionnaire [PHQ‐9]), and assessed outcomes at post‐intervention (8–10 weeks) and follow‐up (22–24 weeks). They received appropriate ethical approval (ref: MS‐IDREC‐C2‐2015‐024 [Espie et al., 2019]; ref MS‐IDREC‐C2‐2014‐034 [Freeman et al., 2017]), and were registered prior to participant enrolment on clinical trial registries. Methods and primary results for both trials have been published elsewhere (Espie et al., 2019; Freeman et al., 2017). The primary trials obtained informed consent from all participants. For this sub‐analysis we selected all those participants who, in addition to probable insomnia disorder, had a PHQ‐9 score ≥ 10 at baseline; this cut‐off being suggestive of clinically significant depressive symptoms (National Collaborating Centre for Mental Health, 2018). We also examined the likelihood participants experienced clinically significant improvement (PHQ‐9 < 10), and reliable change (PHQ‐9 < 10 with change scores ≥ 6) at post‐intervention and follow‐up (National Collaborating Centre for Mental Health, 2018). Baseline measures are presented using summary statistics, with hypothesis testing to check for balance as the original trials were not stratified for the selection criteria of depression caseness as in this study. Statistical analyses were performed using generalized linear models with maximum likelihood estimation with the following fixed effects: treatment, trial, baseline outcome, and an interaction between baseline and trial to allow for a different baseline effect in each trial. For continuous outcomes this was performed using linear models, for remission analyses (PHQ‐9 < 10) and reliable remission (PHQ‐9 < 10 plus a minimum reduction of ≥ 6) using logistic regression models and mediation analysis was performed using parametric structural equation models (StataCorp version 16). See Supporting information for further details on mediation analysis. Moderation was assessed by adding interactions between moderator and treatment to the analysis models. All analyses were based on the intention‐to‐treat principle.

## 3 RESULTS

Participants meeting the depressive symptom threshold (*N* = 3,352) comprised 61.5% of the combined trial sample (*N* = 5,466), ranged in age from 18 to 89 years (mean [*SD*]: intervention 29.6 years [13.0]; control group 29.4 years [12.9]) and were predominantly female (76% in both groups). Insomnia symptoms (SCI‐8: intervention = 8.05 [4.1]; control = 8.04 [4.2]; *t*_3,350_ = −0.04, *p* = .97) and depressive symptoms (PHQ‐9: intervention = 15.3 [4.2]; control = 15.2 [4.3]; *t*_3,350_ = −0.20, *p* = .84) were not significantly different between groups at baseline, and PHQ‐9 scores indicated moderate‐to‐severe depressive symptoms.

**FIGURE 1:**
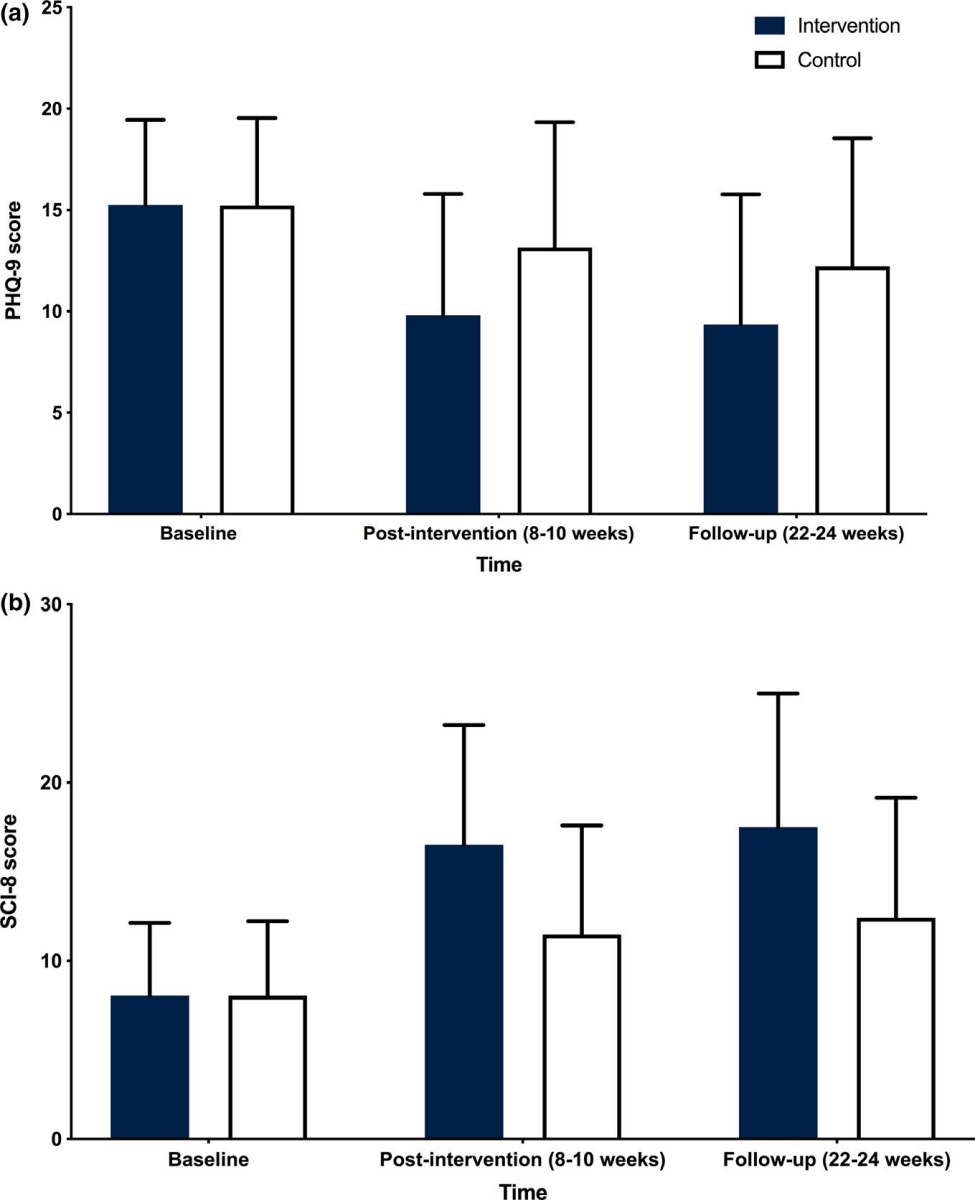
Mean depressive symptoms (a) and insomnia symptoms (b) over time. Note: (a) Average depressive symptoms measured by the 9‐item Patient Health Questionnaire (PHQ‐9). Outcomes were assessed at baseline, post‐intervention (weeks 8 and 10) and follow‐up (weeks 22 and 24). Error bars represent standard deviations. (b) Average insomnia symptoms were measured by the 8‐item Sleep Condition Indicator (SCI‐8). Higher scores on the SCI‐8 indicate better sleep

First, and consistent with published trial results, digital CBT led to significant improvements in insomnia symptoms (SCI‐8) at post‐intervention (8–10 weeks; adjusted mean difference 5.19, 95% CI 4.63−5.75, *g* = 0.76) and follow‐up (22–24 weeks; adjusted mean difference 5.15, 95% CI 4.47−5.83, *g* = 0.69) compared with control. Second, digital CBT also significantly reduced depressive symptoms (PHQ‐9) at post‐intervention (8–10 weeks; adjusted mean difference −3.03, 95% CI −3.56 to −2.50, *g = *0.48) and follow‐up (22–24 weeks; adjusted mean difference −2.74, 95% CI −3.34 to −2.15, *g* = 0.42) compared with control (Figure 1; Table 1). Removing the sleep item from the PHQ‐9 did not alter the effects of digital CBT on depressive symptoms.

**TABLE 1:** Effects of digital CBT versus control (Usual care and Sleep Hygiene Education) on insomnia symptoms (Sleep Condition Indicator [SCI‐8]) and depressive symptoms (9‐item Patient Health Questionnaire [PHQ‐9])

Assessment	Unadjusted, Mean (*SD*)		Adjusted difference (95% CI)	Hedges’ *g*	*p‐*Value
	Control (*n* = 1,656)	Intervention (*n* = 1,696)			
SCI‐8*					
Baseline	8.04 (4.2)	8.05 (4.01)			
Post‐intervention (Weeks 8 and 10)	11.5 (6.1)	16.5 (6.73)	5.19 (4.63, 5.75)	0.76	< .001
Follow‐up (Weeks 22 and 24)	12.42 (6.72)	17.52 (7.48)	5.15 (4.47, 5.83)	0.69	< .001
PHQ‐9					
Baseline	15.2 (4.3)	15.3 (4.2)			
Post‐intervention (Weeks 8 and 10)	13.16 (6.18)	9.81 (5.98)	−3.03 (−3.56, −2.50)	0.48	< .001
Follow‐up (Weeks 22 and 24)	12.22 (6.32)	9.35 (6.42)	−2.74 (−3.34, −2.15)	0.42	< .001

CI, 95% confidence interval; PHQ, Patient Health Questionnaire; SCI‐8, Sleep Condition Indicator; *SD*, standard deviation.

* Higher score indicates less impairment.

In relation to our questions of interest, those receiving digital CBT for insomnia compared with controls were 2.9 times more likely to experience clinically significant improvement at post‐intervention PHQ‐9 < 10 (OR = 2.9; 95% CI = 2.34, 3.65, *p* < .001). This effect was 2.4 times at follow‐up (OR = 2.4, 95% CI = 1.96, 3.14, *p* < .001). They were also 2.9 times more likely to achieve that post‐intervention endpoint with a reliable change in PHQ‐9 (≥ 6 points) (OR = 2.93, 95% CI = 2.4, 3.7, *p* < .001). The comparable statistic was 2.7 times at follow‐up (OR = 2.7, 95% CI = 2.12, 3.37, *p* < .001).

Importantly, in relation to the hypothesis of insomnia improvement as an explanatory factor, mediation analyses demonstrated that improvements in sleep (on the SCI‐8) at mid‐intervention (weeks 3–4) mediated 87% of the intervention effect on depressive symptoms at post‐intervention (weeks 8–10; Table 2). With the sleep item removed from the PHQ‐9, improvements in sleep (SCI‐8) at mid‐intervention mediated 91% of the intervention effect on depressive symptoms at post‐intervention. We also found that intervention effects were not moderated by age, gender, or by baseline insomnia or depressive symptoms.

**TABLE 2:** Mediation analysis results

Assessment	Mediation tested	Total effect		Direct effect		Indirect effect		Mediation (%)
		Effect size (95% CI)	*p*‐Value	Effect size (95% CI)	*p*‐Value	Effect size (95%CI)	*p*‐Value	
PHQ‐9								
Post‐intervention	SCI‐8 mid‐intervention	−2.55 (−3.14, −1.97)	< .001	−0.33 (−0.95, 0.29)	.29	−2.22 (−2.58, −1.87)	< .001	87%

PHQ‐9, Patient Health Questionnaire; SCI‐8, Sleep Condition Indicator.

## 4 DISCUSSION

Cognitive behavioural therapy is already the guideline treatment for insomnia disorder (Riemann et al., 2017). Our findings indicate that the effects of digital CBT for insomnia are not limited to sleep but extend to improving depressive symptoms, in accordance with other literature (Gebara et al., 2018). Indeed, these results suggest that intervention upon sleep may increase the odds of depression improvement (PHQ‐9 < 10) by a factor of 2.4−3 times. This may be a causal relationship, because reduction in insomnia symptoms at mid‐intervention (3–4 weeks) mediated the effect of the intervention on depressive symptoms at post‐intervention (8–10 weeks). The finding that intervention effects were not moderated by demographics or by more severe baseline symptoms of insomnia or depression supports the generalizability of these data. Putative mechanisms proposed to account for the antidepressant effects of CBT for insomnia include sleep restriction therapy and stimulus control increasing slow‐wave sleep, which is known to be disrupted in both depression and insomnia (Krystal & Edinger, 2010; Staner, 2010), thereby increasing homeostatic sleep drive. Additionally, establishment of a consistent sleep−wake schedule and increased time awake during the day may contribute to behavioural activation (Maroti, Folkeson, Jansson‐Fröjmark, & Linton, 2011), and cognitive therapy, when applied to insomnia, may result in generalized benefits to mood through a marked shift in perspective when a fundamental dilemma becomes resolved, consistent with cognitive models of depression (Beck, 2002).

Our results should be interpreted with some caution given that they are derived from only two effectiveness RCTs, and these were recruited samples. However, the sample size was large enough to reliably test these relationships, and the homogeneous nature of the intervention usefully reduces the variance typically associated with combining data across diverse CBT approaches to intervention. Further work is warranted, especially in large clinical samples, to consider sleep as a therapeutic target to improve depressive symptoms, especially in patients with both insomnia disorder and major depressive disorder. Finally, given that population‐level access to therapist‐delivered CBT is limited due to lack of therapists and other barriers (Koffel et al., 2018), digital CBT may offer an accessible first‐step intervention to improve insomnia with attendant clinical benefits to depressive symptoms.

## Conflict of interest

ALH and JRC are employed by Big Health Inc. (Sleepio), receive a salary and are shareholders. CBM, MLD, JK are employed by Big Health Inc. and are salaried by the company. CAE is the Co‐Founder and Chief Medical Officer of Big Health Inc. and is a shareholder. BS offered clinical consultancy to Big Health Inc. during the course of the OASIS trial. RE is a paid consultant of Big Health Inc. AIL was employed by Big Health Inc. when the DIALS trial was being conducted. The digital self‐help intervention was made available to all participants at no cost. No other investigators report conflicts of interest. Both studies were conducted at the University of Oxford, Sleep & Circadian Neuroscience Institute and Department of Psychiatry. The University of Oxford has a Memorandum of Understanding with Big Health Inc. for the conduct of joint research.

## Author contributions

ALH, CBM and CAE conceived the study question. AIL, CAE, DF and BS collected data in the original trials. RE performed the statistical analysis. All authors performed statistical interpretation. ALH, CBM and CAE wrote the paper. All authors critically edited the paper. All authors agreed to submission.

## Supporting information


Supplementary Material
