# Supplementary material for: Insomnia as a mediating therapeutic target for depressive symptoms: A sub‐analysis of participant data from two large randomized controlled trials of a digital sleep intervention
Source: J Sleep Res. 2020 Aug 18;30(1):e13140. doi: 10.1111/jsr.13140 (PMC8150672; doi:10.1111/jsr.13140)
Supplement: Supplementary file 1 — Supplementary Material [file JSR-30-e13140-s001.docx]

**Supporting information**

**Mediation analysis**

Sleep (SCI-8) at mid-intervention (weeks 3 to 4) was considered as a mediator of the effect of digital CBT on PHQ-9 at post-intervention (weeks 8 to 10). The analysis used structural equation modelling by estimating a linear model for the mediator with group assignment, baseline PHQ-9, baseline SCI-8 as covariates, and estimating a linear model for PHQ-9 with SCI-8 at mid-intervention, group assignment, baseline PHQ-9 and baseline SCI-8 as covariates. The effect of group assignment on SCI-8 is multiplied by the effect of SCI-8 on PHQ-9 to estimate the indirect effect, and the effect of digital CBT on PHQ-9 in the model including SCI-8 at mid-intervention is an estimate of the direct effect. The proportion mediated is the indirect effect divided by the total effect. As a sensitivity check, we repeat the analysis using the PHQ-8, having removed the sleep item.
